# When Michaelis and Menten met Holling: towards a mechanistic theory of plant nutrient foraging behaviour

**DOI:** 10.1093/aobpla/plu066

**Published:** 2014-10-22

**Authors:** Gordon G. McNickle, Joel S. Brown

**Affiliations:** 1 Department of Biology, Wilfrid Laurier University, 75 University Avenue West, Waterloo, ON N2L 3C5, USA; 2 Department of Biological Sciences, University of Illinois at Chicago, 845 W. Taylor St. (MC066), Chicago, IL 6060, USA

**Keywords:** Encounter rate, handling time, Holling's disc equation, Michaelis–Menten kinetics, nutrient foraging, plant foraging behaviour, root foraging precision.

## Abstract

Plants are adept at assessing and responding to nutrients in soil, and generally proliferate roots into nutrient-rich patches. An analogy between this growth response and animal foraging movement is often drawn, but because of differences between plants and animals it has not always been clear how to directly apply existing foraging theory to plants. Here we suggest one way to unite pre-existing ideas in plant nutrient uptake with foraging theory. First, we show that the Michaelis–Menten equation used by botanists and the Holling disc equation used by zoologists are actually just rearrangements of the same functional response. This mathematical unity permits the translation of existing knowledge about the nutrient uptake physiology of plants into the language of foraging behaviour, and as a result gives botanists direct access to foraging theory. Second, we developed a model of root foraging precision based on the Holling disc equation and the marginal value theorem, and parameterize it from the literature. The model predicts (i) generally plants should invest in higher quality patches compared to lower quality patches, and as patch background–contrast increases; (ii) low encounter rates between roots and nutrients result in high root foraging precision; and (iii) low handling times for nutrients should result in high root foraging precision. The available data qualitatively support these predictions. Third, to parameterize the model above we undertook a review of the literature. From that review we obtained parameter estimates for nitrate and/or ammonium uptake for 45 plant species from 38 studies. We observe that the parameters ranged over six orders of magnitude, there was no trade-off in foraging ability for nitrate versus ammonium: plants that were efficient foragers for one form of nitrogen were efficient foragers for the other, and there was also no phylogenetic signal in the parameter estimates.

## Abstract (precis)

To the untrained eye plants might appear to be more like an inanimate object than the type of organism that exhibits behaviour. However, if you know where to look, you will find that plants are remarkably good at assessing and responding to environmental conditions in ways that are best described using behavioural models. Here we show that botanists and zoologists have been using rearranged forms of an identical equation to explain resource intake. By rearranging this equation, botanists may preserve existing knowledge on uptake physiology, while simultaneously gaining access to a rich literature on foraging theory.

## Introduction

Nutrients are typically distributed heterogeneously throughout the soil (Jackson and Caldwell 1993; Hutchings and de Kroon 1994; Hodge 2004) and plants are adept at assessing and responding to this nutrient heterogeneity (Robinson 1994; de Kroon and Hutchings 1995; Hodge 2004; Kembel and Cahill 2005; Cahill and McNickle 2011; Tian and Doerner 2013). Generally, plants respond to nutrient-rich patches by preferentially proliferating roots into those patches. This growth response results in an increased absorptive surface area inside nutrient-rich patches relative to lower quality regions of the average background soil and is generally considered to be an adaptive response. Increasingly, there has been a trend towards viewing this plasticity in root growth with respect to nutrients through a lens of behavioural ecology (Sutherland and Stillman 1988; Silvertown and Gordon 1989; Kelly 1992; Hutchings and de Kroon 1994; de Kroon and Hutchings 1995; Gersani *et al.* 2001; Dudley and File 2007; Hodge 2009; Karban 2008; McNickle *et al.* 2009; Cahill and McNickle 2011; Tian and Doerner 2013). This gradual shift in perspectives on how plants acquire nutrients has been driven by data demonstrating that plants are not passively following pre-determined growth trajectories, but instead plant growth is based on actively assessing and responding to cues from the soil nutrient environment (Silvertown and Gordon 1989; Hutchings and de Kroon 1994; Hodge 2009; Karban 2008; Cahill and McNickle 2011; Tian and Doerner 2013).

However, despite substantial gains in our knowledge of the range of plant nutrient foraging behaviours, we are still a long way from incorporating plant foraging behaviour as a united sub-field of behavioural ecology (McNickle *et al.* 2009; Cahill and McNickle 2011). Indeed, many questions remain about plant root foraging behaviour. For example, the average plant appears to strongly proliferate roots into nutrient-rich patches; however, some species do not strongly proliferate roots into patches (Hodge 2004; Kembel and Cahill 2005; Kembel *et al.* 2008; Cahill and McNickle 2011). Additionally, some species of plants appear to discriminate among patches of varying quality by proliferating higher root mass into more nutrient-rich patches, while other species show little discrimination among patches that differ in nutrient availability (Gleeson and Fry 1997; Hutchings *et al.* 2003; McNickle and Cahill 2009). There are also unresolved questions about how plants should invest in patches based on the contrast between nutrient availability in rich patches versus poorer background soil (Lamb *et al.* 2004). Logically, we would expect all plant species to benefit from nutrient-rich patches (when they are nutrient limited) and so we lack a first principles explanation that can permit an understanding of why species differ so much in their foraging responses (Kembel *et al.* 2008; McNickle and Cahill 2009; McNickle *et al.* 2009).

In a perfect world plant foraging theory would not reinvent the wheel, but integrate existing ideas about plant ecology, plant nutrient uptake physiology and behavioural ecology. Here, we attempt such a synthesis by exploring the previously recognized fact that the Holling disc equation used by foraging ecologists to model resource intake (Holling 1959; Stephens and Krebs 1986; Vincent *et al.* 1996; Stephens *et al.* 2007) and the Michaelis–Menten equation used by plant physiologists to model nutrient uptake (Michaelis and Menten 1913; Lineweaver and Burk 1934; Epstein and Hagen 1951; Johnson and Goody 2011) are actually rearranged forms of the same functional response (Real 1977). As we show, this identity in functional response permits the translation of plant nutrient uptake physiology into the language of foraging behaviour. We have three main objectives: first, we compare the models used by biologists to describe resource capture to show that the models used by plant physiologists and animal behaviourists are mathematically identical. Second, we derive a simple example model to predict the root foraging precision of plants that is based on the well-described functional response of plants (Epstein and Hagen 1951; Bassirirad 2000) and the marginal value theorem (Charnov 1976; McNickle and Cahill 2009). Third, we parameterize the foraging model with a realistic range of plant foraging traits obtained from a literature review of existing studies of plant uptake kinetics for nitrate and ammonium and recast these results from ‘enzyme-kinetics’ into ‘foraging kinetics’. We also present a summary of these data with three sub-objectives: (i) we describe the range and central tendency within the observed patterns of nutrient uptake traits; (ii) we ask whether there is any relationship in the ability of plants to capture the substitutable resources of nitrate and ammonium and (iii) we ask whether there is any phylogenetic signal in these uptake parameters. We conclude with a discussion of the value of rethinking plant uptake of nutrients as a process of enzyme kinetics to a process of foraging behaviour.

## Methods

### Identical models, different packaging

**Figure 1.**
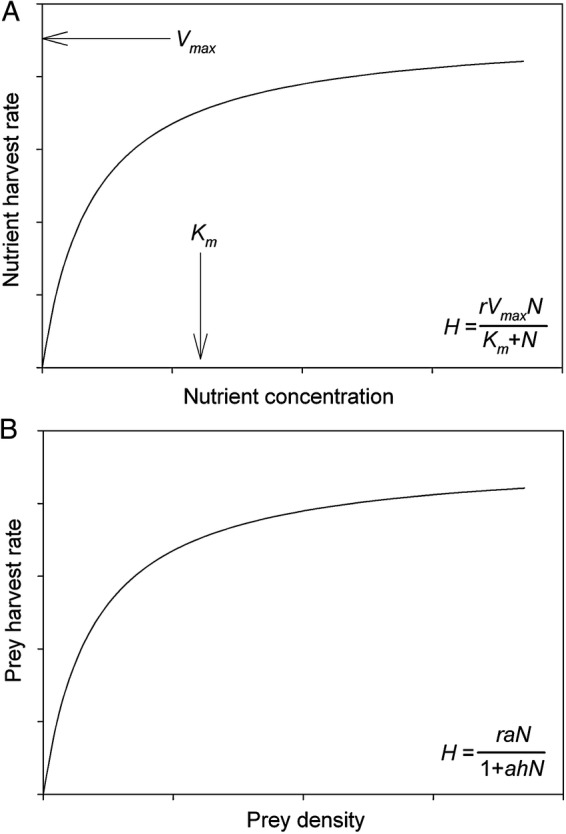
Graphical comparison of (A) the Michaelis–Menten equation that relates resource harvest rates to resource abundance and (B) the Holling disc equation that relates resource harvest to resource abundance. The parameters of the Michaelis–Menten equation describe the shape of the curve where *V*_max_ is the maximum resource harvest rate and *K*_m_ is the concentration of nutrients that produces half of the maximum resource harvest rate (A). The parameters of the Holling disc equation describe traits of the organisms, and though they produce the same curve, these parameters cannot be placed in the figure.

In the broader ecological literature on foraging and forager functional responses, Holling's disc equation (Holling 1959) provides one commonly used framework for modelling resource capture. In the plant literature on nutrient uptake kinetics, the Michaelis–Menten equation (Michaelis and Menten 1913; Lineweaver and Burk 1934; Johnson and Goody 2011) provides the framework for modelling nutrient capture (Epstein and Hagen 1951). Mathematically, these are simply different arrangements of the same functional response, but the arrangements produce distinct interpretations of parameters, and each arrangement naturally lends itself to different predictive objectives (Real 1977). Both equations produce a Type II functional response (*sensu* Holling 1959), where the resource harvest rate, d*H/*d*t*, increases up to an asymptote with resource availability, *N* (Fig. 1), and both have two parameters.

The Michaelis–Menten equation (Michaelis and Menten 1913; Lineweaver and Burk 1934; Johnson and Goody 2011) for nutrient uptake at the level of the whole root system in plants is

(1) $$\frac{dH}{dt}=\frac{rV_{\max}N}{K_{m}+N}$$

where d*H/*d*t* is the resource harvest rate (units of resource uptake per time per gram of root), *r* is the biomass of roots possessed by the plant, *N* is the available nutrient concentration in the environment (units of resources per unit volume of soil), *V*_max_ is the maximum influx rate (units of resources per time per gram of root) and *K*_m_ is the half saturation constant (units of resources per unit volume), representing the resource concentration where the harvest rate is half of the theoretical maximum. Readers should note that *V*_max_ and *K*_m_, simply describe the shape of the functional response (Fig. 1A); the asymptote on the *y*-axis is given by *V*_max*,*_, and the resource concentration on the *x*-axis where the harvest rate is halfway to the asymptote is given by *K*_m_ (Fig. 1A).

Holling's disc equation (Holling 1959) is written as

(2a) $$\frac{dH}{dt}=\frac{aN}{1+ahN}.$$

As above, *dH/dt* is the harvest rate (units of resource per time per individual forager); *N* is the concentration of prey in the environment (more typically referred to as prey abundance, but abundance per area or volume is mathematically identical to the concept of nutrient concentration); *a* is the rate that prey are encountered by the forager (here in units of per time per individual forager, often called search efficiency) and *h* is the time required by the forager to handle each encountered prey item (units of individual forager × time per prey). Note here that instead of describing the shape of the functional response, the parameters *a* and *h* describe activities relevant to the process of foraging.

Typically, Holling's disc equation describes the harvest rate of one animal with one mouth, and so most typically ecologists do not need to clarify that the equation is parameterized on a ‘per-individual forager’ basis as we have done above (McNickle *et al.* 2009). But, recognizing the ‘per unit of forager’ aspect of the equation becomes important when using Holling's disc equation to understand the foraging behaviour of modular plants that are more like colonial animals than solitary animals (see McNickle *et al.* 2009 for discussion). As above, taking into account the per-root foraging effect in plants, equation (2a) becomes

(2b) $$\frac{dH}{dt}=\frac{raN}{1+ahN}.$$

In the root foraging form of the Holling disc equation, a unit of root (*r*, units of mass or length) substitutes for the individual forager, and the plant can effectively be many foragers at once by proliferating many units of root into a volume of soil (McNickle *et al.* 2009).

The parameters of the Michelis–Menten equation can be recast into Holling's disc equation with a simple rearrangement of equation (1). Dividing both sides of the Michaelis–Menten equation by *K*_m_ and setting equal to the disc equation we find

(3) $$\frac{r(V_{max}/K_{m})N}{1+(1/K_{m})N}=\frac{raN}{1+ahN}.$$

Equation (3) shows how to convert the enzyme-kinetic parameters into the foraging parameters where the encounter rate between a unit of root and a nutrient molecule is given by *a* = *V*_max_*/K*_m_, and the cost in time associated with handling a given amount of nutrient molecules is given by *h* = 1/*V*_max_. This translation produces estimates of plant foraging parameters for the Holling disc equation that are in the correct units and maintain the correct theoretical interpretation for plant foraging (Table 1).

**Table 1.** Comparison of the parameters of the Michaelis–Menten equation, and the Holling disc equation. The two models are built from an equation of the same general form (Real 1977) and the two equations model identical processes in plants and animals. This produces interchangeable parameters, where the units of measurement also perfectly translate.

	Parameters	Units	Biological meaning
Holling disc equation	$a=V_{\max}/K_{m}$	L g^−1^ min^−1^	Effective encounter rate or search efficiency; the maximum volume of nutrients of concentration *R* (µmol/L) that are encountered per gram of root per minute.
	$h=1/V_{\max}$	min g µmol^−1^	Handling time: the time taken for 1 µmol of nutrient to be captured by a gram of root
Michaelis–Menten equation	$V_{\max}=1/h$	µmol g^−1^ min^−1^	Maximum theoretical rate of nutrient uptake, per gram of root.
	$K_{m}=1/ah$	µmol L^−1^	Half saturation constant or the nutrient concentration where the rate of uptake is half of *V*_max_. This is sometimes called the enzyme affinity for the substrate.

### From functional responses to root behaviour

From the Holling equation, where parameters map directly to functional behavioural traits, many aspects of foraging behaviour can be intuitively derived as a direct consequence of search and handling (reviewed in Stephens *et al.* 2007). Here we advance a simple nutrient foraging model for plants which is based on Charnov's (1976) marginal value theorem and the Holling disc equation as one example of the value of translating Michaelis–Menten kinetics into Holling's foraging kinetics. The marginal value theorem hypothesizes that foragers should invest effort (here effort is root biomass) into patches until the nutrient uptake rate inside the patch balances the rate in the background soil (Charnov 1976; Gleeson and Fry 1997), and several species of plants have been shown to follow this prediction (Kelly 1990; Kelly 1992; McNickle and Cahill 2009). For plants that can place foraging roots in multiple locations, this prediction is also similar to the ideal free distribution (Fretwell and Lucas 1969; McNickle and Brown 2014). However, plant foraging is sufficiently different from animal foraging that one further modification is necessary.

Plant foraging is often measured as root foraging precision, which compares the investment of root biomass or root length inside a patch with other locations in the soil. This plant foraging behaviour differs from animal foraging where questions are typically about understanding time investment or energy requirements (McNickle *et al.* 2009). Thus, the model we develop predicts root foraging precision, which we define as the ratio of root production inside a nutrient-rich patch of some volume to the root production in the poorer quality background soil of equal volume (e.g. Rajaniemi and Reynolds 2004; James *et al.* 2009; McNickle and Cahill 2009). It is important to note that many other definitions of root foraging precision have been used by empiricists. However, all of these definitions of precision are conceptually similar in that they attempt to estimate the relative investment of root biomass inside a nutrient-rich patch relative to the investment in average background habitat quality. These other definitions are not easily predicted from the functional response without more complicated treatments of root : shoot growth or spatial dimensions of soil. For example, some authors defined precision as the mass of roots inside a patch as a fraction of total body mass (Campbell *et al.* 1991) but a model for this type of foraging precision would require significantly more complex treatments of root growth relative to nutrients and shoot growth relative to photosynthetically active radiation that are beyond the scope of this manuscript. Other authors have defined precision as the relative proportion of total root system inside a patch (e.g. Kembel and Cahill 2005) or the relative root mass difference between patch and background as a fraction of total root biomass (e.g. Einsmann *et al.* 1999). These are also difficult to solve without complex and explicit treatments of space at the scale of the entire root system that are beyond the goals of this manuscript.

Consider a plant searching for *j* forms of nitrogen (*N_ij_*) spread across *i* patches throughout the soil. The total amount of nitrogen *j* encountered is given by the search efficiency for nitrogen types *j* to *n* (*a_j_ … a_n_*), the concentration of nitrogen types *j* to *n* at location *i* in the soil (*N_ij_ … N_in_*) and the amount of roots that are searching in each location *i* (*r_i_ … r_k_*). Uptake rate is discounted by the rate at which resources are encountered while searching (*a_j_* … *a_n_*), and by the time lags associated with handling nitrogen type *j* to *n* (*h_j_ … h_n_*). Assuming that *N_ij_* is experimentally held constant over the course of the experiment then with no depletion, (e.g. Campbell and Grime 1989; Shemesh *et al.* 2010), and also assuming that the concentrations of nutrients other than nitrogen are experimentally held constant among locations (e.g. Drew and Saker 1975; McNickle *et al.* 2013), then the harvest rate of nitrogen types *j* to *n* at location *i* is given by the multiple resource form of the Holling disc equation, with root biomass:

(4) $$\frac{dH_{i}}{dt}=\frac{r_{i}\left(\sum_{\;j=1}^{n}a_{j}N_{ij}\right)}{1+\left(\sum_{\;j=1}^{n}a_{j}h_{j}N_{ij}\right)}.$$

Equation (4) predicts the uptake rate of all nitrogen types from any location *i*. Consider a simple experiment where plants are grown with one spatially discrete nutrient-rich patch (location *p*), surrounded by nutrient-poor background soil (location *b*). Comparing one patch with a similar volume of background soil, we expect that, all else equal, plants would produce roots in the patch (*r_p_*) and in the background (*r_b_*), such that the amount of roots combine with the traits of the forager (*a* and *h*) to produce equal rates of nutrient uptake (Charnov 1976), given by

(5a) $$\frac{r_{p}\left(\sum_{\;j=1}^{n}a_{j}N_{\;pj}\right)}{1+\left(\sum_{\;j=1}^{n}a_{j}h_{j}N_{\;pj}\right)}=\frac{r_{b}\left(\sum_{\;j=1}^{n}a_{j}N_{bj}\right)}{1+\left(\sum_{\;j=1}^{n}a_{j}h_{j}N_{bj}\right)}.$$

Here we are interested in root foraging precision, which is the optimal ratio of roots inside the patch relative to the roots inside the background soil (P* = r_p_*/r_b_*) given by:

(5b) $$P^{*}=\frac{r_{p}^{*}}{r_{b}^{*}}=\frac{\sum_{\;j=1}^{n}N_{\;pj}}{1+\sum_{\;j=1}^{n}a_{j}h_{j}N_{\;pj}}\frac{1+\sum_{\;j=1}^{n}a_{j}h_{j}N_{bj}\;\;}{\sum_{\;j=1}^{n}N_{bj}}.$$

Equation (5b) thus represents a simple approximation of foraging precision in plants based on plant nutrient uptake physiology and foraging theory. A key assumption of this model is that plants are nitrogen limited and not limited by other resources, particularly carbon. When plants are carbon limited they may shift allocation away from roots and towards shoot production. Additionally, this model assumes that the roots are the sole source of nitrogen uptake. For example, root production may not be as important for nitrogen acquisition in nitrogen-fixing plants or mycorrhizal species. These assumptions can be easily met in controlled manipulative experiments and by choosing appropriate model species, but may not apply to all species and contexts.

### Literature review: range of foraging traits

To estimate the range of behavioural foraging traits in plants and parameterize our model, we broadly searched the literature for estimates of *V*_max_ and *K*_m_ for nitrate and ammonium and translated the reported *V*_max_ and *K*_m_ into encounter rates and handling times (Table 1). In February 2011, we searched the ISI Web of Science for the topic ‘root uptake kinetics’ which returned 870 papers. To make search results more manageable, we filtered the results to the Web of Science Category ‘*Plant Sciences*’. This produced 509 papers. We then inspected titles and abstracts to reduce the search to only papers that reported parameters for nitrate and/or ammonium. From the remaining 219 papers we read each manuscript to collect parameter estimates. We limited our data collection to papers that estimated parameters based on either fresh or dry weight of roots and that estimated both *V*_max_ and *K*_m_ using the Michaelis–Menten equation. Despite the fact that all plant papers we reviewed used the two-parameter Michaelis–Menten equation to fit their data, a surprisingly large number of papers only reported *V*_max_, while failing to report the second parameter, *K*_m_*.* We excluded these papers. Additionally, we limited the data to only plant species with areal shoots so that nutrient capture was achieved exclusively through roots. Fully aquatic plants and algae were therefore excluded, but wetland plants were included. A small number of studies (<10) were not in English, were unavailable after an exhaustive physical and online search or did not report the units of measurement, and these were excluded. When different studies reported estimates of *V*_max_ and *K*_m_ for the same species, we report these as separate data points [**see Supporting Information**]. When multiple treatments were employed we used only the control treatment or the equivalent ‘no manipulation’ treatment. These search criteria resulted in a final set of 38 studies, with parameter estimates for nitrate and/or ammonium for 45 distinct plant species, and three species that had been studied more than once [**see Supporting Information]**.

Parameter estimates were adjusted uniformly to µmol g^−1^ min^−1^ for *V*_max_ and µM for *K*_m_. Parameter estimates from fresh weight and dry weight of roots were plotted and interpreted separately. We used linear regressions to compare foraging ability for nitrate and ammonium (R Statistical environment, R Development Core Team 2009). To summarize the taxonomic diversity and patterns in these parameter estimates, we performed a phylogenetic analysis. The hypothesized phylogenetic relationships among species were constructed using the online phylogenetic database and assembly tool, Phylomatic (Webb and Donoghue 2005), with Phylomatic tree version R20120829 as the backbone for our phylogenetic hypotheses. The Phylomatic tree is well resolved up to the level of family, but the tool places all genera as polytomies within family and all species as polytomies within genera (Kembel and Cahill 2005; Webb and Donoghue 2005). We tested for a phylogenetic signal in the foraging trait data (a and h) by calculating a *K* statistic using the R library ‘Picante’ (Kembel *et al.* 2010). The *K* statistic compares the observed phylogenetic signal in the trait with the signal that would be expected under the Brownian motion model of trait evolution. *K* values >1 imply a strong phylogenetic signal, *K* values equal to 1 imply the Brownian motion model and *K* values <1 imply a random or convergent pattern of evolution. Traits were mapped onto the phylogeny for visualization using the ‘plotBranchbyTrait’ tool in the R library ‘phytools’. For several species there were multiple independent estimates of traits. In these cases, we took the average trait value. Traits were ln(*x* + 1) transformed for normality and to control for differences between fresh and dry weight estimates. There was considerable variation in the methods used among studies to estimate nitrogen foraging parameters [**see Supporting Information**]. Thus, we envision the phylogenies as a way of summarizing the data with respect to taxonomy, but urge caution in interpreting the phylogenetic signal from these data.

## Results

### Range of reported uptake parameters

**Figure 2.**
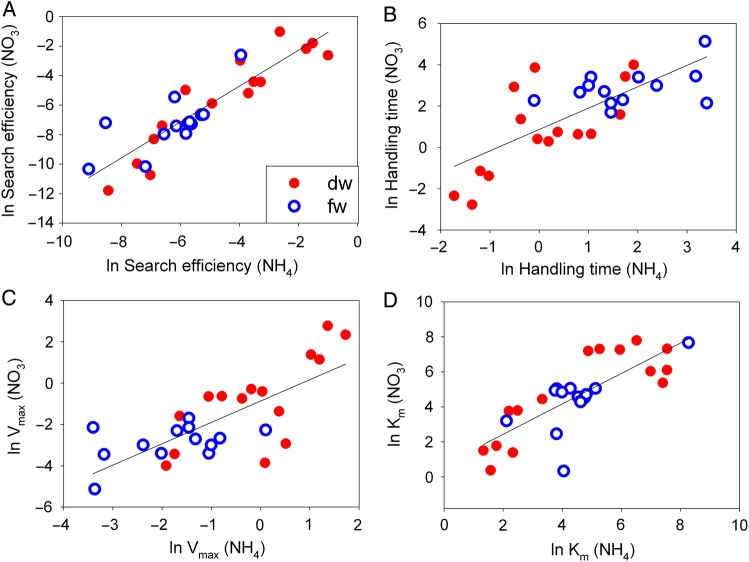
Scatter plots and linear regressions of the observed relationship between (A) the search time for nitrate and ammonium (*F*_1,26_ = 100.4, *P* < 0.0001, *R*^2^ = 0.79), (B) the handling time for nitrate and ammonium (*F*_1,26_ = 31.2, *P* < 0.0001, *R*^2^ = 0.53), (C) *V*_max_ for nitrate and ammonium (*F*_1,26_ = 31.2, *P* < 0.0001, *R*^2^ = 0.53) and (D) *K*_m_ for nitrate and ammonium (*F*_1,26_ = 43.2, *P* < 0.0001, *R*^2^ = 0.61). Fresh (fw) and dry (dw) weights are plotted separately, but the patterns were qualitatively similar and so they were pooled for regression fits. Data were ln(*x* + 1) transformed.

Parameter estimates ranged over six orders of magnitude (Table 2), but were relatively evenly spaced along this range (Fig. 2). Examining the Holling parameters, the minimum value for per gram of root *encounter rate* for nitrate was two orders of magnitude lower than the minimum value for ammonium, while the maximum, mean, mode and coefficient of variation were all similar between nitrate and ammonium (Table 2). The pattern was retained whether the estimate was based on fresh or dry weight of tissue. Again, note that this is the encounter rate between active uptake sites and not the encounter rate of nutrients and the surface of the root.

**Table 2.** Summary of observed parameter estimates from the literature review for plant uptake of nitrate and ammonium. Authors sometimes calculated based on dry or fresh weight of roots, these are summarized independently. Note that *a* and *h* were calculated from *V*_max_ and *K*_m_ according to Table 1.

	Statistic	Dry weight		Fresh weight	
		*a*	*h*	*a*	*h*
NO_3_	Min	7.57E−06	0.062	1.11E−06	5.455
	Max	0.360	54.545	0.074	952.381
	Mean	0.051	11.241	0.005	94.288
	Median	0.011	1.901	0.001	16.300
	CV	0.133	0.135	0.062	0.087
NH_4_	Min	2.16E−04	0.178	1.12E−04	0.902
	Max	0.368	19.690	0.081	30.000
	Mean	0.049	3.191	0.009	10.874
	Median	0.011	2.150	0.003	5.454
	CV	0.109	0.157	0.111	0.259
	**Statistic**	**Dry weight**		**Fresh weight**	
		***K*_m_**	***V*_max_**	***K*_m_**	*V*_max_

NO_3_	Min	1.45	0.0183	1.4	0.001
	Max	2422	16.001	2140	0.183
	Mean	480.3	2.206	205.8	0.0696
	Median	44.0	0.526	75.6	0.062
	CV	0.662	0.545	0.435	1.365
NH_4_	Min	2.3	0.0508	8.3	0.033
	Max	1908	5.633	3930	1.108
	Mean	293.1	1.052	332.03	0.246
	Median	27.8	0.465	72	0.183
	CV	0.507	0.778	0.333	0.904

For *handling time* the maximum and mean estimates were generally larger for nitrate compared with ammonium, while other statistics were relatively similar, or showed no clear pattern (Table 2). Again the pattern was retained whether the estimates were based on fresh or dry tissue. The range of parameter values suggests that nitrate may be more difficult or costly to transport across root membranes compared with ammonium given the higher average handling costs. It also suggests that at the extreme, the number of encounters which turn into effective encounters (i.e. uptake) may be lower for nitrate.

We also present the range of estimates of parameters for the Michaelis–Menten equation. The parameter *V*_max_ is simply the inverse of handling time, and so the patterns in *V*_max_ were the same as for *h* above, but inverted. That is, where handling times were larger for nitrate compared with ammonium, maximum influx rates (*V*_max_) tended to be lower for nitrate compared with ammonium. For the half saturation constant, *K*_m_, there seemed to be no obvious differences between ammonium and nitrate (Table 2). If nutrient uptake in plants is a foraging process, then this may not be surprising since *K*_m_ is actually a combination of search and handling time, and the patterns described above for each of these are cancelled by confounding them within this parameter (i.e. *K*_m_ = 1/*ah*).

We also explored the relationships in nutrient uptake ability for nitrate and ammonium within a species to examine whether plants might specialize in one type of nitrogen over the other (Fig. 2). Here, all four parameters (*a*, *h*, *V*_max_ and *K*_m_) tell a similar story: plants that are efficient foragers for nitrate are also efficient foragers for ammonium. Interestingly, this suggests that there are no general trade-offs in foraging ability for these two common forms of nitrogen, and instead species are either efficient or inefficient foragers. However, note that it is not possible to be simultaneously good at searching and handling because of the way that these parameters are conceptualized (Table 1).

**Figure 3.**
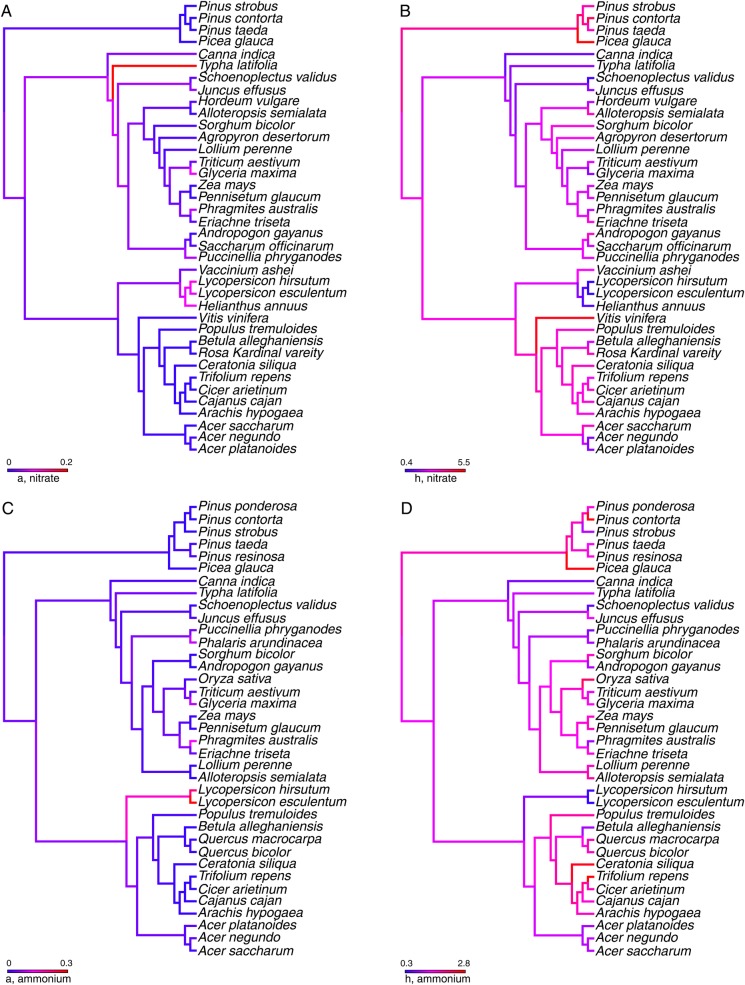
Phylogeny of species for which we have foraging parameters for nitrate (A and B) or ammonium (C and D). Species come from three major clades including conifers, monocots and eudicots. Colour on the branch tips represent ln(*x* + 1) transformed trait values for search efficiency (A and C) and handling time (B and D). The species lists for nitrate and ammonium foraging parameters were not identical and so the upper and lower phylogenies are not identical.

In our phylogenetic analysis of trait values, we found no evidence of any phylogenetic signal for any of the foraging traits (Fig. 3). The foraging traits for nitrate encounter rates (*K* = 0.23, *P* = 0.032; Fig. 3A) or handling (*K* = 0.17, *P* = 0.011; Fig. 3B) and ammonium encounter rates (*K* = 0.15, *P* = 0.161; Fig. 3C) or handling (*K* = 0.09, *P* = 0.625; Fig. 3D) all had a *K* statistic <1 implying random evolution of traits, or convergent evolution towards relatively uniform foraging traits across all taxa. At this time we are unable to determine whether this result reflects a true lack of historical relationships, or merely the variability in methodologies used to estimate parameters and urge caution in interpreting this signal.

### Root foraging precision

**Figure 4.**
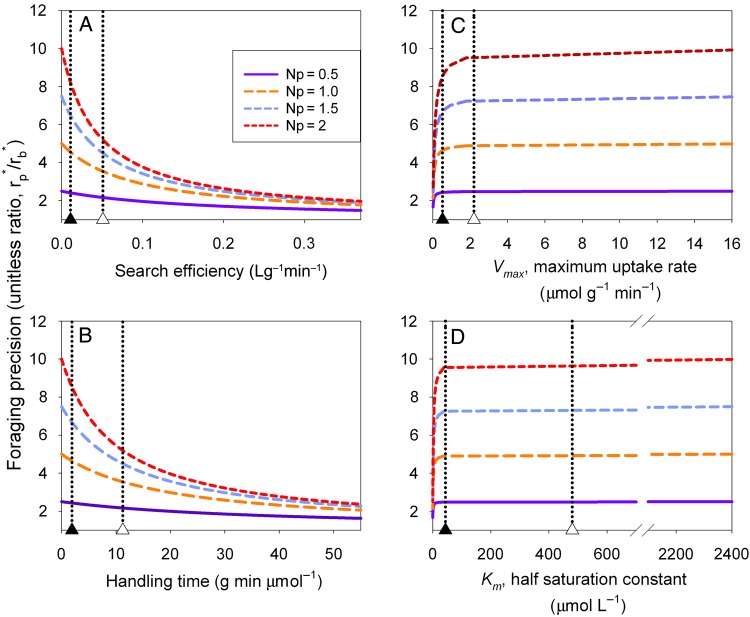
Predicted root foraging precision over the observed range of search efficiency (A), handling time (B), maximum uptake rate (C) and half saturation constant (D) among plants. In each case the value of the background soil was *N*_b_ = 0.2 µmol L^−1^, and the value of the patch associated with each curve is shown in the figure legend (0.5 µmol L^−1^ < *N*_p_ < 2 µmol L^−1^). In each panel, the *x*-axis parameter was varied, while the second parameter was held constant at the mean observed value for nitrate calculated from dry weight (Table 2). In each panel, the mean (open triangle) and median (closed triangle) observed values of parameters are indicated by dotted vertical lines.

Using the observed range of parameter values for nitrate (Table 2), we can generate hypotheses concerning the corresponding hypothesized range of foraging precision among species foraging for nitrate and how these relate to foraging traits of plants (Equation 5b). The model predicts precision between positive infinity (i.e. all roots inside the patch) and one (i.e. no discrimination between patch and background). In most regions of parameter space, and regardless of which uptake model is used (Michaelis–Menten or Holling), root foraging precision is hypothesized to increase with increasing patch quality (Fig. 4). The model predicts that plants should allocate more roots to increasingly nutrient-rich patches relative to the average poorer quality background soil; that is with increasing patch–background contrast. Each parameter has specific links to predicted foraging behaviour (Fig. 4), and in the following paragraphs we examine each of the model parameters and their hypothesized effect on root foraging precision individually.

Within the range of *a_j_* observed for plants (Table 2), the marginal value theorem hypothesizes that plants possessing the ability to encounter nitrogen at a high rate should discriminate less among patches of differing quality compared with plants with low encounter rates. Theoretically this occurs because encounter rate acts like a scaling parameter for the effectiveness of each unit of root produced. Plants with high encounter rates between uptake sites and nutrient ions will be able to gather more resources, with a low investment in root surface area compared with plants with low encounter rates that require high amounts of surface area to effectively encounter nutrients. We observe from our examination of the literature that the mean and median plant species in our dataset of 45 species possess relatively low values of this parameter compared with the maximum range that is observed in the literature (Table 2, Fig. 4A). Thus, based on our literature review parameterized model the marginal value theorem would predict that the average plant should have relatively high root foraging precision, and should discriminate among patches of different quality by putting more roots in higher quality patches. However, the literature review also reveals that species exist with extremely efficient encounter rates, and these species are predicted to exhibit low root foraging precision in any patch and exhibit similar root foraging precision regardless of patch quality.

In the context of handling time (Fig. 4B), plants with low handling times have low time lags associated with nutrient uptake, meaning that within a prescribed amount of time spent foraging for nutrients (e.g. the duration of a foraging experiment), plants with low handling times are able to actually acquire more of the available nitrogen compared with plants with high handling time. Logically, plants with low handling times for nitrogen are hypothesized to have high nutrient foraging precision because plants with low handling times are able to capitalize on high quality patches more than plants with high handling costs. As above, we observe from our literature review that the mean and median plant species in our dataset of 45 species possess relatively low handling time compared with the range observed in the literature (Table 2). This means that as above, the average plant in our dataset of 45 species has relatively high foraging precision, and discriminates a great deal among patches of differing quality. However, the observed range for handling time is large, suggesting that some species are hypothesized to have relatively low foraging precision, and not discriminate among patches of variable quality (Fig. 4B).

Finally, in the context of either parameter of the Michaelis–Menten equation (Fig. 4C and D), only the lowest values of *V*_max_ or *K*_m_ lead to much discrimination among species with respect to root foraging precision. For either parameter, we see that a plateau is reached quite quickly, and then root foraging precision changes only slightly. Mathematically, this happens because *V*_max_ is the inverse of handling time, and *K*_m_ is the inverse and the product of both search and handling. Roughly, this reverses the patterns observed for the Holling parameters. Biologically, it suggests that the concepts of maximum uptake rate (*V*_max_) and the half saturation constant (*K*_m_) are simply not concepts that are particularly informative about processes important for root proliferation and nutrient foraging. Instead, as we have shown, ecologists interested in nutrient foraging behaviour of plants will be able to discriminate more clearly among the behaviours and traits of plants by translating the Michaelis–Menten parameters into Holling parameters (Fig. 4, Table 1).

## Discussion

We had three major objectives in this paper: First, we showed that the Michaelis–Menten and Holling equations are mathematically identical, and how to translate parameter estimates for each model back and forth (Table 1). The most important insight from this exercise is that under a foraging interpretation of nutrient uptake, the parameter *K*_m_ turns out to be a confounded mixture of search and handling that is not particularly useful for predicting plant foraging behaviour (Fig. 4). The equality of the Michaelis–Menten and Holling equations is mathematically straightforward, and we do not discuss this further.

### Empirically derived parameter estimates

The analysis of the parameter estimates themselves yielded interesting insights. Interestingly, within species, their ability to capture ammonium and nitrate was positively correlated for all parameters (Fig. 2). This means that there are no trade-offs in the ability of species to capture these two important nitrogen types; instead there are ‘super-foragers’ where some species are extremely efficient either encountering or handling both nitrate and ammonium simultaneously, while other species are extremely inefficient. Given that these parameters ranged over six orders of magnitude, this ‘super-forager’ effect is very large indeed (Table 2). However, it should be noted that there is a trade-off (not shown) between encounter rate and handling time. We do not show this because it is a necessary mathematical condition on the way *a* and *h* are calculated (see Table 1). But it is important to note that while plants can be simultaneously efficient at either encountering or handling nitrate and ammonium, they cannot be simultaneously efficient at both encountering and handling. Thus, species must specialize on one or the other of these foraging processes, and ‘super-foragers’ should actually be unpacked into ‘super-encounterers’ versus ‘super-handlers’ where species cannot be both. It remains unclear what forces cause this specialization, and our phylogenetic analysis did not shed any light on this problem.

We analysed the parameter data in the context of historical phylogenetic relationships among species, and the results of the phylogenetic analysis were consistent with random or convergent evolution on foraging traits. However, we suggest that convergent evolution is unlikely since the parameter values varied over six orders of magnitude instead of converging on a single value. We are reluctant to draw too many conclusions about this result because of the large differences in methods among studies from which we obtained parameter estimates. Currently we are inclined to suspect that the observed lack of phylogenetic signal may simply reflect this diversity of methods. We suggest caution in interpreting this result at this time, but this is certainly a pattern that demands further investigation using a common set of methods and a large taxonomic sample.

### A model of root foraging precision

The foraging model generated three predictions: (i) on average, plants should invest more root biomass into higher quality patches relative to lower quality patches but not all species should be expected to discriminate among patches of differing quality depending on their foraging traits (Fig. 4); (ii) root foraging precision, and discrimination among patches of variable quality, should be lowest in species with high encounter rates between nutrients and active uptake sites (Fig. 4A); and (iii) root foraging precision and discrimination among patches of variable quality should be lowest in plants with the highest handling times for nutrients (Fig. 4B).

Empirically testing these hypotheses will require studies that include a large number of taxonomically diverse plant species and produce paired estimates of physiological uptake parameters and root foraging precision (e.g. to generate an empirically derived version of Fig. 4). Kembel and Cahill (2005) assembled a dataset of root foraging precision for ∼120 species. Unfortunately, there are only five species in common between the precision dataset from Kembel and Cahill (2005) and the assembled dataset of uptake parameters presented here. A second problem is that Kembel and Cahill (2005) defined precision as the percentage of total roots inside a patch which is not predicted from the model presented here. Thus, currently there are not enough data available to quantitatively test the foraging model. However, we note that, qualitatively, the available data support the model. For example, the model predicts based on data we assembled from the literature that the average plant should have relatively high root foraging precision, and indeed high root foraging precision appears to be the behaviour of the average plant species (Hutchings and de Kroon 1994; Hodge 2004; Kembel and Cahill 2005; Cahill and McNickle 2011). Further, the prediction that some species should strongly discriminate among patches of variable quality while others should not discriminate among patches is also supported by the available data (Fransen and de Kroon 2001; Hutchings *et al.* 2003; Lamb *et al.* 2004; McNickle and Cahill 2009).

There has been no clear explanation for why some plants should exhibit high root foraging precision while others should not (Robinson 1994; Robinson 1996; Kembel *et al.* 2008), or why some species discriminate among patches of variable quality while others do not (McNickle and Cahill 2009; McNickle *et al.* 2009). Indeed, some authors have even gone so far as to suggest that the large range of behaviour of root proliferation is illogical (Robinson 1994, 1996) or that certain results suggest the behaviour might even be maladaptive (Fransen and de Kroon 2001) when explained using previous conceptual frameworks. We suggest that recasting enzyme kinetics as a foraging process of search and handling provides one clear first principles hypothesis for why so many plant species exhibit high root foraging precision (Fig. 4). Low encounter rates and low handling times intuitively lead to high root foraging precision by virtue of the marginal value theorem (Fig. 4). However, there is a theoretical trade-off between search and handling: foragers cannot do both simultaneously leading to differences in adaptation and ultimately foraging behaviour (Holling 1959).

Switching from the Michaelis–Menten enzyme kinetics view of nutrient uptake to Holling’s functional response view of nutrient uptake as foraging behaviour will require integration of some new concepts into our understanding of plant nutrient foraging. Since handling time is just the inverse of the maximum uptake rate (*h* = 1/*V*_max_) then handling time, as a concept, is already in common use by plant biologists. Plants with high influx rates necessarily have low handling times. However, as we have shown, switching to the inverse of *V*_max_ allows a more subtle discrimination between the foraging behaviour of different species with differing uptake abilities (Fig. 4B versus C) and as a result is a more ecologically valuable parameter.

The concept of encounter rate, which can also be thought of as search efficiency (Stephens *et al.* 2007), is a relatively new idea for plant ecologists that was confounded, along with handling time, inside the half saturation constant (*K*_m_ = 1/*ah*). The concept of encounter rates is critically important in the foraging literature, and is important for understanding the patch-use behaviour of foragers (Vincent *et al.* 1996; Stephens *et al.* 2007). Just as the inverse of influx rate is a more informative parameter for root foraging behaviour, unpacking encounter rate from within *K*_m_ provides better insights into root foraging behaviour of plants that was obscured inside of *K*_m_ (Fig. 4A versus D). Encounters will be influenced by any factor that influences the rate at which nutrient ions are encountered by active uptake sites on a plant root and can include behavioural responses of the plant such as changing the number of active uptake sites (Lauter *et al.* 1996; McNickle *et al.* 2009), or by changing total root biomass/length and therefore the number of active uptake sites per volume of soil (Hutchings and de Kroon 1994; Cahill and McNickle 2011). Encounter rate will also be influenced by physical properties of the soil and physical properties of the nutrient molecules that might limit ion movement in soil solution. For example, most uptake studies are conducted in nutrient solutions within laboratories, which likely have quite high mobility of ions leading to artificially high encounter rates. However, physical factors that limit diffusion rates in field soil will also limit the rate at which plants can encounter nutrients and should have an influence on plant foraging behaviour. An experimental test of the root foraging precision model could manipulate encounter rates by manipulating properties of the soil environment. For example, soils with high clay content have lower mobility of cations, and this would limit the ability of plants to encounter positively charged ions such as nitrate.

With any model there are caveats around the assumptions made. We assumed that nitrogen was the only limiting resource. This is unlikely to be true in many contexts, but can and has been achieved in controlled experiments (e.g. Drew and Saker 1975; McNickle *et al.* 2013). Mathematically, the model could be extended to include foraging for multiple essential resources by the use of a minimum function, where foraging decisions were based on Liebig's law of the minimum. This would require a more complex model, but foraging theory exists for this problem (See Vincent *et al.* 1996; Simpson *et al.* 2004). Additionally, we assumed that nitrogen levels were not depleted over the course of the experiment. Again, controlled manipulative experiments can and have met this assumption (e.g. Campbell and Grime 1989; Shemesh *et al.* 2010). This assumption could be relaxed by allowing nutrients to have their own dynamics in the model (see Vincent *et al.* 1996). Relaxing both of these assumptions would change the quantitative predictions of our model, but we do not believe they would change the qualitative predictions. Specifically, that encounter rates and handling times are important predictors of foraging precision. The assumptions that plants are limited by nitrogen more than carbon can be more easily met by ensuring adequate light supplies. Similarly, the assumption that uptake is achieved through roots alone can be met by the selection of model species, or by sterilizing soil prior to experimentation. Finally, this model assumes that foraging is all that matters to plants. There are many other problems such as mutualisms, enemy attack and competition that plants must solve (De Deyn and Van der Putten 2005; McNickle and Dybzinski 2013), and trade-offs required to solve these problems may cause undermatching in foraging behaviour as plants direct resources away from nutrient foraging and towards solving these other problems (Brown 1988; Nonacs 2001).

The model of root foraging precision presented here is just one example of how the application of a Holling functional response to plant nutrient uptake could enhance our understanding of plant nutrient foraging behaviour, and we hope this work will lead to further advances. For example, much of the existing foraging theory in the animal literature is based upon the Holling disc equation (Real 1977; Stephens and Krebs 1986; Vincent *et al.* 1996; Stephens *et al.* 2007; Abrams 2010*a*, 2010*b*) and a diversity of models can be derived from this functional response. We suggest that interested plant ecologists can now begin to take full advantage of the existing foraging literature by using the Holling equation to interpret nutrient uptake instead of the Michaelis–Menten equation. It is beyond the scope of this manuscript to review the existing animal models (see Stephens and Krebs 1986; Stephens *et al.* 2007), but we believe that there is considerable room for enhanced linkages between processes of interest to plant ecologists and plant physiologists mediated through pre-existing understanding of foraging ecology.

## Conclusions

We have argued that a switch from the phenomenological view of plants as passive *enzyme-like* entities that are largely governed by chemical fluxes to a more mechanistic view of plants as active foragers that assess and respond to their environment fits with the trend towards viewing plant plasticity as a behavioural process. We believe that the ability to translate existing plant physiological data into information relevant to foraging behaviour and theory will be valuable for plant ecologists. Our model has the potential to generate improved ecological understanding by uniting traditionally separate fields of ecology, while still preserving our existing knowledge and understanding.

## Sources of Funding

Our work was funded by a Natural Sciences and Engineering Research Council (Canada) Post-Doctoral Fellowship and a Banting Post-Doctoral Fellowship to G.G.M.

## Contributions by the Authors

G.G.M. and J.S.B. conceived the study and developed the theoretical approach. J.S.B. developed the comparison between the Holling and Michaelis–Menten equations. G.G.M. developed the root foraging model, performed the literature review and analysed the models. Both authors contributed to the writing of the manuscript.

## Conflicts of Interest Statement

None declared.

## Supporting Information

The following Supporting Information is available in the online version of this article –

**File S1.** Text with two figures. The Holling disc equation and Michaelis–Menton equation were fit to the same randomly generated data to demonstrate that they are mathematically identical.

**File S2.** Supporting data. The Michaelis–Menten parameter estimates collected from the literature for (i) nitrate, (ii) ammonium including (iii) metadata, and (iv) reference list.

Additional Information
