# Supplementary material for: When Michaelis and Menten met Holling: towards a mechanistic theory of plant nutrient foraging behaviour
Source: AoB Plants. 2014 Oct 22;6:plu066. doi: 10.1093/aobpla/plu066 (PMC4271705; doi:10.1093/aobpla/plu066)
Supplement: Additional Information [file supp_6_plu066_index.html]

When Michaelis and Menten met Holling: towards a mechanistic theory of plant nutrient foraging behaviour — Additional Information 

# When Michaelis and Menten met Holling: towards a mechanistic theory of plant nutrient foraging behaviour

## Additional Information

Additional Information

**Files in this Data Supplement:**

- Additional Information - xlsx file
